# Prognostic factors and models to predict pediatric sepsis mortality: A scoping review

**DOI:** 10.3389/fped.2022.1022110

**Published:** 2023-02-23

**Authors:** Irene Yuniar, Cut Nurul Hafifah, Sharfina Fulki Adilla, Arifah Nur Shadrina, Anthony Christian Darmawan, Kholisah Nasution, Respati W. Ranakusuma, Eka Dian Safitri

**Affiliations:** ^1^ Department of Child Health, Dr. Cipto Mangunkusumo Hospital, Faculty of Medicine, University of Indonesia, Jakarta, Indonesia; ^2^ Clinical Epidemiology and Evidence-Based Medicine Unit, Dr. Cipto Mangunkusumo Hospital, Faculty of Medicine, University of Indonesia, Jakarta, Indonesia

**Keywords:** sepsis, scoring, PELOD, PRISM, mortality, prediction

## Abstract

**Introduction:**

Several scoring systems are available to assess the severity of sepsis in pediatric patients in diverse settings worldwide. This study investigates the quality and applicability of predictive models for determining pediatric sepsis mortality, especially in acute care and limited-resource settings.

**Data sources:**

Mortality prediction factors and models were searched in four databases using the following criteria: developed for pediatric health care, especially in acute settings, and with mortality as an outcome.

**Study selection:**

Two or more reviewers performed the study selection to ensure no bias occurred. Any disagreements were solved by consensus or by the decision of a third reviewer.

**Data extraction:**

The authors extracted the results and mapped the selected studies qualitatively to describe the prognostic properties of the risk factors and models proposed in the study.

**Data synthesis:**

The final analysis included 28 mortality prediction models. Their characteristics, analysis, and performance measures were summarized. Performance was described in terms of calibration and discrimination, including assessing for risk of bias and applicability. A modified version of the PRISM-III score based on physiologic criteria (PRISM-III-APS) increased its predictive value to 0.85–0.95. The vasoactive-inotropic score at 12 h had a strong independent association with death. Albumin had an excellent predictive value when combined with other variables. Lactate, a biomarker widely measured in patients with sepsis, was highly associated with mortality. The bioimpedance phase angle was not considered applicable in our setting. Measurement using more straightforward methods, such as mid-upper arm circumference, was feasible in numerous health care facilities.

**Conclusion:**

Leveraging prognostic models to predict mortality among pediatric patients with sepsis remains an important and well-recognized area of study. While much validation and development work remains to be done, available prognostic models could aid clinicians at the bedside of children with sepsis. Furthermore, mortality prediction models are essential and valuable tools for assessing the quality of care provided to critically ill pediatric patients.

## Introduction

Sepsis is a systemic inflammatory response syndrome triggered by infections caused by various pathogens, resulting in severe sepsis and septic shock (1). Sepsis remains a significant cause of morbidity, mortality, and high health care costs in the pediatric population worldwide (2). In the United States, the incidence of severe sepsis was 5.16 per 1,000 infants (1). In children in pediatric intensive care units (PICU) in developing countries, the sepsis mortality rate is higher than 50% (2). The World Health Organization has estimated that sepsis causes 4 million deaths per year worldwide in children under five years old (3).

Several scoring systems are available to assess the severity of sepsis in pediatric patients (4–6). However, these systems were created across many different settings worldwide; they therefore might not be ideal for pediatric patients with sepsis in developing countries or in otherwise resource-limited settings. Thus, a scoring model that can assess pediatric sepsis in a stratified manner is needed to guide physicians in promptly treating these patients, particularly in acute care settings during the initial stages of sepsis (5, 7, 8). Although sepsis is one of the leading causes of mortality in hospitalized patients, information regarding predictive factors for mortality and morbidity is limited (2–4, 7, 9–13).

As a preliminary step, we searched for existing reviews of predictive factors and models to predict pediatric sepsis mortality in several databases and search platforms, such as PubMed, Cochrane Central, ProQuest, PROSPERO, the WHO Trial Registry, the Clinical Trial Registry, and Google Scholar, and did not find any similar studies. Therefore, we conducted this scoping review to provide a comprehensive, systematic overview of the various predictive models and scores available to guide clinicians in managing pediatric sepsis. This study investigates the quality and applicability of predictive models for assessing pediatric sepsis mortality, especially in acute care and resource-limited settings.

## Methods

The main objective of the present review was to synthesize the evidence associated with broad research topics and to identify the forms of evidence available. The flexibility of this review method allowed us to broadly explore and incorporate different study designs. Although quality assessment is not included in this review, the methodology applied to synthesize this knowledge is systematic and thereby accessible for critical analysis.

This study explored the literature on the prognostic models available between 2010 and 2020 to predict pediatric sepsis mortality. In developing this study, we used the checklist of review processes from the Preferred Reporting Items for Systematic Reviews and Meta-Analyses extension for Scoping Reviews (PRISMA-ScR). The framework of this study consists of several steps, including (1) identifying the research question, (2) identifying inclusion and exclusion criteria, (3) developing a search strategy and selecting evidence, (4) extracting and analyzing data, and (5) presenting the results.

### Stage 1: Research question

The diversity among the available scoring systems for predicting sepsis mortality could lead to late diagnosis or misdiagnosis, thus potentially increasing the mortality rate. The research question was developed in consideration of this problem. The primary question was formulated by incorporating the Population, Concept, and Context (PCC) elements. Our primary question was “What prognostic models are available for predicting mortality in pediatric patients with sepsis?” and the sub-question was the application of those prognostic models in acute care, particularly in the crucial initial phases when decisions about further treatment must be made promptly, and in the context of resource-limited settings.

### Stage 2: Inclusion and exclusion criteria

**Figure 1:**
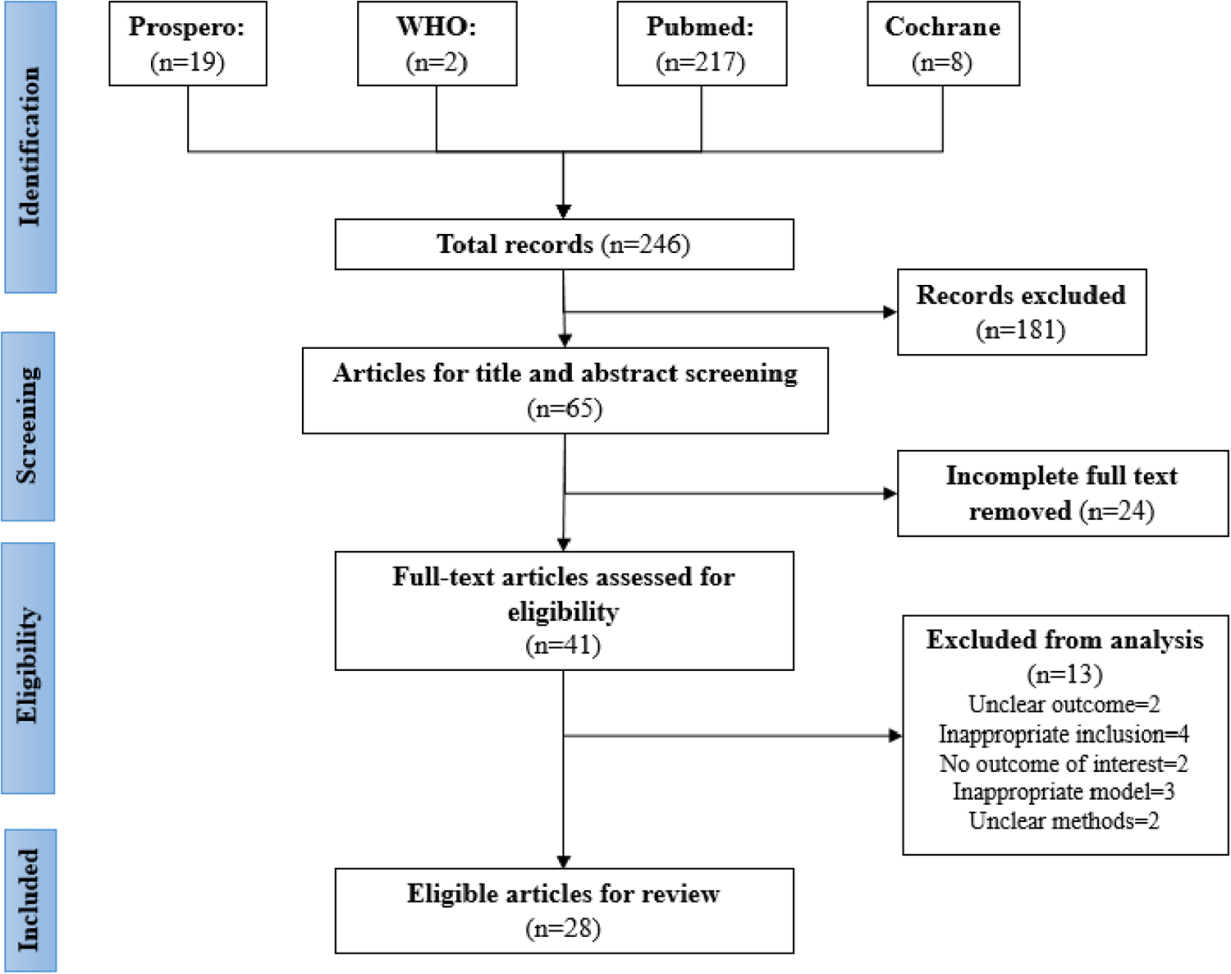
Flow Diagram of Search.

The inclusion and exclusion criteria for this study are shown in Figure 1. Evidence was selected based on the inclusion criteria, with the selection performed by two or more reviewers to ensure no bias occurred. Any disagreements were solved by consensus or by the decision of a third reviewer. The reviewers conducted their screening according to the PRISMA-ScR checklist. Ineligible papers were eliminated.

### Stage 3: Search strategy and evidence selection

We searched MEDLINE (PubMed), PROSPERO, EMBASE, ProQuest, the WHO Registry, the Clinical Trial Registry, and the Cochrane Library using a tailored search strategy to identify all the relevant titles and abstracts of studies published in English between January 2010 and December 2020 that discussed predictive/prognostic scores or models that could be used in the management of sepsis. The main keywords in the search strategy were “prognostic” OR “predictive” OR “prognosis,” coupled with (AND) “model” OR “score,” coupled with (AND) “sepsis” OR “septic shock” OR “severe sepsis,” coupled with (AND) “pediatric” OR “paediatric” OR “child” OR “infant,” and excluding (NOT) “neonate” OR “neonates.” Gray literature was obtained by identifying similar articles in the references of eligible articles.

We excluded editorials, case studies, conference abstracts, unpublished studies, and expert commentaries. For studies with more than one publication of findings, we selected the most recent publication. We also excluded studies that contained models or scores aimed at diagnosing sepsis. We intended to limit the scope of the study to only those models that could be used to predict severity, mortality, or risk of complications. Three independent reviewers screened the titles and abstracts to ensure compliance with the inclusion and exclusion criteria mentioned above and settled any conflicts by mutual agreement.

### Stage 4: Data extraction and analysis

The three independent reviewers used data extraction sheets that were prepared before screening to obtain the following details for inclusion in the final review: last name of the first author; date of publication; period of patient recruitment and follow-up; country of study; aims/purpose; sample size; age group; methodology; type of predictive model; the name of the model; and outcomes and how they were measured. The authors extracted the results and mapped the selected studies qualitatively to describe the prognostic properties of the models used to predict mortality as proposed in their respective studies.

### Stage 5: Presentation of the results

We presented our results in a table to clarify which prognostic models are adequate to predict mortality rates in pediatric sepsis. This table also helped in identifying gaps where further studies are needed.

## Results

The selection of the source of evidence is described in Figure 1. Out of the 246 articles selected from four databases, 181 duplicate articles were excluded. Then, during screening, approximately 24 articles that had incomplete full text were removed. Articles that had unclear (two articles) or no (two articles) outcome of interest, inappropriate prediction models (three articles), inappropriate inclusion criteria (four articles), or unclear methods (two articles) were also excluded. The final review included 28 eligible articles, comprising nine studies with a single predictor, twelve studies with prognostic models [e.g., pediatric risk of mortality (PRISM), disseminated intravascular coagulation (DIC) score, vasoactive-inotropic score (VIS), pediatric logistic organ dysfunction (PELOD) score, or pediatric sequential organ failure assessment (pSOFA) scor], and seven studies that investigated the performance of a single predictor mixed with available predictor models. Tables 2–6 presents a comparison of the included studies.

**Table 1:** Characteristics of the mortality prediction models.

Study no.	Mortality prediction model	Year published	Development database	Data assembly period	PICU population	Outcome		Hospital mortality rate in each development setting	Data collection	Handling of missing data
						Primary	Secondary			
1	Bioelectrical impedance phase angle Zamberlan et al. (14)	2019	247	One-year period Prospective	Age 2 months—18 years, Brazil	30-day mortality PICU LOS		14.6%	During 30 days of hospitalization	Not explained
2	Troponin T, PELOD 2 Dauhan et al. (15)	2019	41	October 2017–March 2018 Prospective	Age 1 month − ≤ 18 years, Medan, North Sumatera	PICU Mortality		61%	The worst value within the first 24 h and 48 h after PICU admission	Not explained
3	Crystalloid fluid administration over 3 days Zhang et al. (16)	2018	79	2011–2016 Retrospective	Patients admitted to PICU, Chongqin, China	PICU mortality	PICU LOS	35.4%	High fluid administration within three days of PICU stay	Excluded
4	Serum albumin Kim et al. (17)	2017	431	January 1, 2012–December 31, 2015 Retrospective	Age 1 month–18 years, Seoul, Republic of Korea	28-day mortality		19.49%	The worst value within 24 h of PICU admission	No missing data
5	Day-1 PELOD-2 and day-1 “quick” PELOD-2 (qPELOD-2), pSOFA, *P*-MODS Zhong et al. (18)	2019	516	June 2016–June 2018 Retrospective	Age 1 month–14 years, Zhanjiang City, Guangdong Province, China	In-hospital mortality		5.4%	Worst value after 24 h of admission	Excluded
6	A mortality risk model for pediatric sepsis Chen et al. (2)	2017	788	January 2012–June 2014 Retrospective	Age 1 month–14 years, Hunan, China	In-hospital mortality		26.6%	The worst value within 24 h after admission	Excluded
7	PRISM, PRISM III, PRISM IV, PIM, PIM2, PIM3, PELOD, PELOD 2 Niederwanger et al. (19)	2020	398	2000–2019 Retrospective	Age < 18 years, Innsbruck, Austria	In-hospital mortality and MODS		13.6%	Worst value on the day of admission and the day of peak CRP	Analyzed separately and compared
8	Lactate level Jat et al. (20)	2011	30	One year study period Prospective	Age 1 month–12 years, New Delhi, India	Mortality		34.8%	The worst value within 24 h after admission	Excluded
9	Vasoactive-inotropic score McIntosh et al. (21)	2017	138	January 2012–June 2015 Retrospective	Age 60 days–18 years, Taiwan	Ventilator days, ICU length of stay	Composite outcome of cardiac arrest/ECMO/in-hospital mortality	6%	The worst value within 48 h post ICU admission	Excluded
10	DIC score Slatnick et al. (22)	2020	1,653	April 1, 2012–June 26, 2017 Prospective	Age 60 days–18 years, Colorado, USA	Requirement of vasopressors	48-h mortality, ventilator requirement, PICU admission, hospital LOS	2.1% (30-day), 3.5% (90-day), 8.1% (1-year)	Worst value within 24 h ED admission	Excluded
11	PRISM score El-Nawawy (23)	2003	406	March 1, 2000–March 31, 2001 Prospective	Patients admitted to the PICU in El-Shatby Children's Hospital, Egypt	PICU mortality		50.5%	The worst value within 8 h after PICU admission	Not explained
12	Serum procalcitonin, serum albumin, PEWS Xie et al. (24)	2019	205	October 2015–December 2017 Prospective	Age 6 months–9 years, Hubei, China	Mortality		23.4%	The worst value within 24 h after admission	No missing data
13	pSOFA score El-Mashad et al. (25)	2020	281	March–November 2018 Prospective	Age 1 month–18 years in two PICUs, Egypt	30-day mortality	PICU LOS	28.1%	Within 24 h following PICU admission	No missing data
14	PRISM, PELOD El-Hamshary et al. (26)	2017	237	January–December 2011 Retrospective	Patients admitted to the PICU, Cairo, Egypt	PICU mortality		40%	The worst value within 24 h after admission	Not explained
15	Immunology markers Ibrahiem et al. (27)	2016	57	March–December 2014 Prospective	Patients admitted to the PICU, Cairo, Egypt	Mortality		64%	Worst value at days 1 and 7 following PICU admission	No missing data
16	High-sensitivity C-reactive protein, serum procalcitonin, pancreatic stone protein Wu et al. (28)	2017	214	March 2014–October 2015 Prospective	Patients with sepsis admitted to the PICU, Hubei, China	28-day mortality		36.9%	During 28-day hospitalization	Missing data not clearly analyzed
17	Reduction in procalcitonin level Poddar et al. (29)	2016	25	March 2011–June 2013 Prospective	Age ≤ 18 years and admitted to the ICU, India	28-day mortality		55%	On day 1 and day 4 of hospitalization	No missing data
18	Age-adjusted quick SOFA Van Nassau et al. (5)	2017	864	March 2013–January 2018 Retrospective	Age < 18 years, Netherlands	Composite of PICU transfer and mortality	Prolonged LOS	2.7%	The worst value within 24 h of admission	Excluded
19	Plasma mtDNA level Yan et al. (30)	2018	123	July 2013–December 2014 Prospective	Patients admitted to PICU, Hunan, China	In-hospital mortality		21.1%	Within 1st hour of hospital admission	No missing data
20	Urinary L-FABP Yoshimatsu et al. (31)	2016	126	April 2010–December 2011 Prospective	Age 6 months–59 months, admitted to ICU, Dhaka, Bangladesh	Mortality		21%	On day 1 and day 2 after ICU admission	Excluded
21	Thrombomodulin Khattab et al. (32)	2020	140	October 2018–September 2019 Prospective	Age 1 month–18 years, admitted to PICU, Egypt	In-hospital mortality or 30-day mortality following hospital discharge	PICU LOS, hospital LOS, duration of ventilator	7.1%	Within 24 h of PICU admission	No missing data
22	Vascular reactivity index Lee et al. (33)	2021	33	2003–2007 Retrospective	Age < 18 years, Taiwan	28-day mortality		54.5%	Within the first 72 h following PICU admission	No missing data
23	Modified PRISM-III Leon et al. (34)	2005	171	Periods of data assembly not specified Prospective	Age 1 month–16 years, admitted to PICU, Leon, Mexico	Mortality		24.7%	Within 8-h following PICU admission	No missing data
24	Thiol-disulphide homeostasis Ayar et al. (35)	2019	78	March 2015–February 2016 Prospective	Age 3 months–18 years, Turkey	Mortality		28.9%	Within 72 h after being diagnosed with sepsis/septic shock	No missing data
25	Macrophage migration inhibitory factor Emonts et al. (36)	2007	77 children, 68 adults	Periods of data assembly not specified Prospective	Patients admitted to the PICU, Rotterdam, Netherlands	Mortality		13%	Within 24 h following PICU admission	No missing data
26	PRISM-III-APS Pollack et al. (37)	1997	11,163	1989–1994 Retrospective	Patients admitted to the PICU at 32 study sites, Washington, DC, USA	24-h mortality		4.8%	The worst value within 24 h following PICU admission	No missing data
27	Age-adapted SOFA Wu et al. (38)	2019	1831	January 2009–December 2017 Prospective	Age 1 months—18 years, Guangzhou, China	In hospital-mortality	Mortality or PICU LOS ≥ 7	9.4%	The worst value within 24 h after ICU admission	Input as normal value
28	Metabolomics approach Mickiewicz et al. (39)	2013	146	Periods of data assembly not specified Prospective	Age 1 week–11 years, Canada	PICU mortality, septic shock		6.9%	The timing of data collection was not specified	Excluded

PICU, pediatric intensive care unit; PELOD, performance of the pediatric logistic organ dysfunction; PRISM, pediatric risk of mortality; PIM, Pediatric Index of Mortality; ICU, intensive care unit; pSOFA, pediatric sequential organ failure assessment; mtDNA, mitochondriql DNA; L-FABP, liver-type fatty acid binding protein; MODS; multiple organ dysfunction syndromes; CRP, C-reactive protein; PPV, positive predictive value; NPV, negative predictive value; AUROC, area under the receiver operating characteristic curve.

**Table 2:** Analysis of mortality prediction models.

Study no.	Mortality prediction model	Analysis			
		ROC curve	Association	Correlation	Survival analysis
1	Bioelectrical impedance phase angle Zamberlan et al. (14)	PA, cut-off 2.8° AUC: 0.65; 95% CI, 0.58–0.71 Sensitivity 37.1%, Specificity 86%			
2	Troponin T and I, PELOD 2 Dauhan et al. (15)	Troponin T, cut-off 40.3 ng/ml AUC: 86.4%; 95% CI, 0.75–0.97; *p* < 0.001 Sensitivity 76%, specificity 75% Troponin I, cut-off 0.125 ng/ml AUC: 92.6%; 95% CI, 0.85–1.0; *p* < 0.001 Sensitivity 80%, specificity 81.3%		Troponin T-24 h: *r* = 0.137; *p* = 0.394 Troponin T-48 h: *r* = 0.771; *p* < 0.001 Troponin I-24 h: *r* = 0.326; *p* = 0.037 Troponin I-48 h: *r* = 0.691; *p* < 0.001	
3	Crystalloid fluid administration over 3 days Zhang et al. (16)		High crystalloid with PICU mortality (*p* < 0.041)		
4	Serum albumin Kim et al. (17)	Albumin: AUC 0.702; 95% CI, 0.633–0.772 PIM 3 + albumin: AUC 0.82; 95% CI, 0.766–0.874 PRISM III + albumin: AUC 0.857; 95% CI, 0.81–0.904	Hypoalbuminemia with 28-day mortality rate (*p* < 0.001)		
5	Day-1 PELOD-2 and day-1 “quick” PELOD-2 (qPELOD-2), pSOFA, P-MODS Zhong et al. (18)	PELOD-2, cut-off: 6.5 AUC: 0.916; 95% CI, 0.888–0.938 qPELOD-2, cut-off: 1 AUC: 0.802; 95% CI, 0.765–0.836 pSOFA, cut-off: 7.5 AUC: 0.937; 95% CI, 0.913–0.957 *P*-MODS, cut-off: 3 AUC: 0.761; 95% CI, 0.722–0.798			
6	Mortality risk model for pediatric sepsis Chen et al. (2)	Training group, cut-off: 0.22462 AUC: 0.854l; 95% CI, 0.826–0.881 Sensitivity 85.7%, specificity 70.1% Validation group, cut-off: 0.189165 AUC: 0.844; 95% CI, 0.816–0.873 Sensitivity 87.3%, specificity 67.7%	BNP > 7.1: OR, 1.996; 95% CI, 1.45–2.747; *p* < 0.001 Albumin < 3.5: OR, 1.649; 95% CI, 1.098–2.477; *p* = 0.016 Total bilirubin > 6: OR, 2.3; 95% CI, 1.45–3.658; *p* < 0.001 D-dimer positive: OR, 2.921; 95% CI, 2.078–4.108; *p* < 0.001 Mechanical ventilation over 24 h: OR, 8.272; 95% CI, 5.434–12.592; *p* < 0.001 Lactate >2: OR, 1.556; 95% CI, 1.061–2.282; *p* < 0.024		
7	PRISM, PRISM III, PRISM IV, PIM, PIM2, PIM3, PELOD, PELOD 2 Niederwanger et al. (19)	PRISM: AUC 0.6; 95% CI, 0.49–0.72 PRISM III: AUC 0.74; 95% CI, 0.65–0.82 PRISM IV: AUC 0.69; 95% CI, 0.59–0.79 PIM: AUC 0.76; 95% CI, 0.67–0.85 PIM2: AUC 0.75; 95% CI, 0.66–0.85 PIM3: AUC 0.71; 95% CI, 0.6–0.82 PELOD: AUC 0.69; 95% CI, 0.58–0.8 PELOD2: AUC 0.73; 95% CI, 0.62–0.83 PRISM CRP: AUC 0.66; 95% CI, 0.54–0.79 PRISM III CRP: AUC 0.81; 95% CI, 0.73–0.89 PRISM IV CRP: AUC 0.8; 95% CI, 0.72–0.88 PIM CRP: AUC 0.77; 95% CI, 0.67–0.87 PIM2 CRP: AUC 0.77; 95% CI, 0.67–0.87 PIM3 CRP: AUC 0.73; 95% CI, 0.61–0.85 PELOD CRP: AUC 0.69; 95% CI, 0.58–0.79 PELOD2 CRP: AUC 0.84; 95% CI, 0.77–0.91			
8	Lactate level Jat et al. (20)	PRISM III score, cut-off 10 AUC: 0.909; 95% CI, 0.802–1.016; *p* < 0.0001 PPV 70%, NPV 90% Lactate 1 (0 h–3 h), cut-off 5 mmol/L AUC: 0.786; 95% CI, 0.596–0.975; *p* = 0.014 PPV 38%, NPV 80% Lactate 2 (12 h), cut-off 5 mmol/L AUC: 0.792; 95% CI, 0.597–0.986; *p* = 0.012 PPV 71%, NPV 83%, Lactate 3 (24 h), cut-off 5 mmol/L AUC: 0.786; 95% CI, 0.580–0.991; *p* = 0.023 PPV 64%, NPV 83%	PRISM III score, cut-off 10 OR, 21; 95% CI, 2.155–204.614; *p* = 0.002 Lactate 1 (0 h–3 h), cut-off 5 mmol/L OR, 6.7; 95% CI, 1.047–42.431; *p* = 0.034 Lactate 2 (12 h), cut-off 5 mmol/L OR, 12.5; 95% CI, 1.850–84.442; *p* = 0.005 Lactate 3 (24 h), cut-off 5 mmol/L OR, 8.6; 95% CI, 1.241–61.683; *p* = 0.021		
9	Vasoactive-inotropic score McIntosh et al. (21)			Correlation analysis with ICU LOS and ventilator days	
10	DIC score Slatnick et al. (22)	1-year mortality, cut-off: 3; AUC: 0.69 Sensitivity 0.7, specificity 0.62	30-day mortality: OR, 2.99; 95% CI, 0.54–16.6; *p* = 0.21 90-day mortality: OR, 3.57; 95% CI, 0.90–14.09; *p* = 0.07 1-year mortality: OR, 3.72; 95% CI, 1.48–9.35; *p* = 0.005		1-year mortality: HR 3.55; 95% CI, 1.46–8.64; *p* = 0.005
11	PRISM score El-Nawawy (23)				The cut-off point of survival was a PRISM score of 26 with an expected/observed ratio of 1.05 for non-survivors, with 91.6 percent accuracy.
12	Serum procalcitonin, serum albumin, PEWS Xie et al. (24)	PCT, cut-off: 59.65 mcg/L AUC: 0.73 Sensitivity 53.2%, specificity 85.1% Albumin, cut-off: 3.52 g/dl AUC: 0.761 Sensitivity 57.45%, specificity 85.11% PEWS, cut-off 6.5 points AUC: 0.771 Sensitivity 74.5%, specificity 68.1% PCT, Albumin, PEWS combination AUC: 0.908 Sensitivity 87.23%, specificity 85.11%			
13	pSOFA score El-Mashad et al. (25)	pSOFA, cut-off 6.5 AUC: 0.886; 95% CI, 0.84–0.931; *p* < 0.0001 Sensitivity 80.9%, specificity 81.8%			
14	PRISM, PELOD El-Hamshary et al. (26)	PRISM III, cut-off: 20 AUC: 0.726; 95% CI, 0.661–0.790 Sensitivity 63.8%, specificity 67.1% PELOD, cut-off: 13 AUC: 0.788; 95% CI, 0.729–0.846 Sensitivity 70.2%, specificity 69.9%			
15	Immunology markers Ibrahiem et al. (27)	NK cell concentration, cut-off 10 AUC: 0.95; 95% CI, 0.889–1.0; *p* < 0.001 Sensitivity 100%, specificity 86%, PPV 70%, NPV 100%, accuracy 89.5%			
16	High-sensitivity C-reactive protein, serum procalcitonin, pancreatic stone protein Wu et al. (28)	hsCRP, cut-off: 76.1 mg/ml AUC: 0.76; 95% CI, 0.70–0.82; *p* < 0.01 Sensitivity 87.3%, specificity 60.7% PCT, cut-off 47 ng/ml AUC: 0.83; 95% CI, 0.77–0.88; *p* < 0.01 Sensitivity 72.1%, specificity 68.1% PSP, cut-off 256 ng/L AUC: 0.73; 95% CI, 0.67–0.79; *p* < 0.01 Sensitivity 79.7%, specificity 57.7% PCT, CRP, PSP: AUC 0.92; 95% CI, 0.87–0.95; *p* < 0.001 Sensitivity 73.4%, specificity 93.3%			
17	Reduction in procalcitonin level Poddar et al. (29)	The number of deaths was too small to provide a good estimate of the area under the ROC curve for a reduction in PCT level to predict survival. However, an absolute decrease of PCT of ≥ 4 ng/ml or a percentage reduction of ≥ 50% in the first four days of ICU stay predicted survival with a sensitivity of 78% and specificity of 83%			
18	Age-adjusted quick SOFA Van Nassau et al. (5)	qSOFA score, cut-off: 2 AUC: 0.72; 95% CI, 0.57–0.86 Sensitivity 50, specificity 93.3%, NPV 98%, PPV 22.5%			
19	Plasma mtDNA level Yan et al. (30)	Plasma mtDNA, cut-off: 890.43 AUC: 0.726; *p* < 0.0001 Sensitivity 88.5%, specificity 53.6%			
20	Urinary L-FABP Yoshimatsu et al. (31)	L-FABP first urine, cut-off: 370 ng/ml AUC: 0.663; 95% CI, 0.455–0.871 Sensitivity 75%, specificity 66.7% L-FABP day 2, cut-off: 580 ng/ml AUC: 0.809; 95% CI, 0.612–1,0 Sensitivity 81.8%, specificity 90% L-FABP first urine, cut-off: 2275 mcg/g creatinine AUC: 0.675; 95% CI, 0.463–0.886 Sensitivity 75%, specificity 66.7% L-FABP day 2, cut-off 1,570 mcg/g creatinine AUCL 0.85; 95% CI, 0.666–1,0 Sensitivity 90.9%, specificity 85%			
21	Thrombomodulin Khattab et al. (32)	Thrombomodulin, cut-off: 5.0 AUC: 0.711; 95%CI, 0.569–0.847; *p* = 0.118 Sensitivity 80%, specificity 80%, PPV 24%, NPV 98%, accuracy 80% PRISM, cut-off 4.25 AUC: 0.918; 95% CI, 0.819–1.0; *p* = 0.002 Sensitivity 80%, specificity74%, PPV 69%, NPV 98%, accuracy 74% PIM, cut-off: 6.8 AUC: 0.96; 95% CI, 0.91–1.0; *p* = 0.001 Sensitivity 100%, specificity 86%, PPV 88%, NPV 100%, accuracy 87%			
22	Vascular reactivity index Lee et al. (33)	VRI-24 h, cut-off: 50 AUC: 0.83; *p* = 0.007 Sensitivity 82%, specificity 75%, LR+ 3.3, LR- 0.2; Youden index 0.6 VRI-48 h, cut-off: 61 AUC 0.81; *p* = 0.033 Sensitivity 71%, specificity 82%, LR+ 2.9, LR−0.3; Youden index 0.5			
23	Modified PRISM-III Leon et al. (34)	Modified PRISM-III score, cut-off: 13 Sensitivity 71%, specificity 64%			
24	Thiol-disulphide homeostasis Ayar et al. (35)				
25	Macrophage migration inhibitory factor (MIF) Emonts et al. (36)		MIF levels were significantly higher in non-survivors At entry, *p* < 0.001 At 12 h, *p* = 0.005 At 24 h, *p* = 0.01		
26	PRISM-III-APS Pollack et al. (37)	PRISM III-APS training AUC: 0.95 ± 0.007 PRISM III-APS validation AUC: 0.902 ± 0.027			
27	Age-adapted SOFA Wu et al. (38)	In-hospital mortality, cut-off: 2 Crude AUROC: 0.757; 99%CI, 0.715–0.789; *p* < 0.001 Adjusted AUROC: 0.771; 99% CI, 0.732–0.81; *p* < 0.011			
28	Metabolomics approach Mickiewicz et al. (39)	Metabolomics AUC: 0.91; sensitivity 80%, specificity 90%, PPV 89%, NPV 82%, accuracy 85% PRISM III-APS AUC: 0.85; sensitivity 70%, specificity 80%, PPV 78%, NPV 73%, accuracy 75% Orthogonal partial least squares discriminant analysis models 1st model: septic shock specimens AUC 0.91, *p* = 0.0044 2nd model: septic shock specimen with a complicated course AUC 1.0, *p* = 0.00043			

ROC, receiver operating characteristic; AUC, area under the curve; CI, confidence interval; PICU, pediatric intensive care unit; PELOD, performance of the pediatric logistic organ dysfunction; pSOFA, pediatric sequential organ failure assessment; OR, odds ratio; PRISM, Pediatric Risk of Mortality; PRISM III-APS, Pediatric Risk of Mortality-III-Acute Physiology Score; PIM, Pediatric Index of Mortality; PIM, Pediatric Index of Mortality; DIC, disseminated intravascular coagulation; HR, hazard ratio; PEWS, Pediatric Early Warning Score; L-FABP, liver-type fatty acid binding protein; PA, phase angle; MODS, multiple organ dysfunction syndromes; CRP, C-reactive protein; PPV, positive predictive value; NPV, negative predictive value; AUROC, area under the receiver operating characteristic curve.

**Table 3:** Performance of mortality prediction model.

Mortality prediction model	Risk of bias				Acceptability			Overall	
	Participant	Predictors	Outcome	Analysis	Participant	Predictor	Outcome	Risk of bias	Applicability
Bioelectrical impedance phase angle Zamberlain et al. (14)	+	+	+	−	+	+	+	−	+
Troponin-T, PELOD 2 Dauhan et al. (15)	−	+	+	−	+	+	+	−	+
Crystalloid fluid administration over 3 days Zhang et al. (16)	+	+	+	−	−	+	+	−	−
Serum albumin Kim et al. (17)	−	+	+	−	+	+	+	−	+
Day-1 PELOD-2 and day-1 “quick” PELOD-2 (qPELOD-2), pSOFA, P-MODS Zhong et al. (18)	−	+	+	−	+	+	+	−	+
Mortality risk model for pediatric sepsis Chen et al. (2)	−	+	+	−	−	+	+	−	−
PRISM, PRISM III, PRISM IV, PIM, PIM2, PIM3, PELOD, PELOD 2 Niederwanger et al. (19)	−	+	+	−	+	+	+	−	+
Lactate level Jat et al. (20)	+	+	+	−	+	+	+	−	+
Vasoactive-inotropic score McIntosh et al. (21)	+	+	+	−	+	+	+	−	+
DIC score Slatnick et al. (22)	+	+	+	−	+	+	+	−	−
PRISM score El-Nawawy (23)	+	+	+	+	−	+	+	+	−
Serum Procalcitonin, serum albumin, PEWS Xie et al. (24)	+	+	+	−	+	+	+	−	+
pSOFA score El-Mashad et al. (25)	+	+	+	−	+	+	+	−	+
PRISM, PELOD El-Hamshary et al. (26)	−	+	+	−	−	+	+	−	−
Immunology markers Ibrahiem et al. (27)	+	+	+	−	+	+	+	−	+
High-sensitivity C-reactive protein, serum procalcitonin, pancreatic stone protein Wu et al. (28)	+	+	+	+	+	+	+	−	−
Reduction in procalcitonin level Poddar et al. (29)	+	+	+	−	+	+	+	−	+
Age-adjusted quick SOFA Van Nassau et al. (5)	−	+	+	−	−	+	+	−	−
Plasma mtDNA level Yan et al. (30)	−	+	+	−	+	−	+	−	−
Urinary L-FABP Yoshimatsu et al. (31)	+	−	+	−	+	−	+	−	−
Thrombomodulin Khattab et al. (32)	+	+	+	−	−	−	+	−	−
Vascular reactivity index Lee et al. (33)	−	+	+	−	+	+	+	−	+
Modified PRISM-III Leon et al. (34)	+	+	+	−	−	+	+	−	−
Thiol-disulphide homeostasis Ayar et al. (35)	−	+	+	−	+	−	+	−	−
Macrophage migration inhibitory factor (MIF) Emonts et al. (36)	+	−	+	−	−	+	+	−	−
PRISM-III-APS Pollack et al. (37)	+	+	+	−	−	+	+	−	−
Age-adapted SOFA Wu et al. (38)	+	+	+	−	−	+	+	−	−
Metabolomics approach Mickiewicz et al. (39)	+	+	+	−	−	-	+	−	−

PELOD-2, performance of the pediatric logistic organ dysfunction; PRISM, pediatric risk of mortality; PIM, Pediatric Index of Mortality; DIC, disseminated intravascular coagulation; pSOFA, pediatric sequential organ failure assessment; L-FABP, L-FABP, liver-type fatty acid binding protein.

**Table 4:** Characteristics of included studies (*n* = 28).

	*n* (%)
Publication year	
1990–2000	1 (3.6)
2001–2010	3 (10.7)
2011–2021	24 (85.7)
Economic status of included country(ies)	
Single country	14 (50)
Lower-middle income	4 (28.6)
Upper-middle income	4 (28.6)
High income	6 (42.8)
Country not specified	0
Study design	
Interventional (e.g., RCT)	0
Randomized (e.g., cluster RCTs)	0
Observational (e.g., cross-sectional)	0
Prospective cohort	19 (61)
Retrospective cohort	11 (39)
Secondary research (e.g., review)	0
Predictor	
Single predictor	12 (46)
Prognostic models	9 (29)
Mixed	7 (25)
Health care settings	
In-hospital setting	15 (52)
Paediatric intensive care unit	13 (48)
Analysis approach	
Quantitative	28 (100)
Qualitative	0

RCT, randomized controlled trial.

**Table 5:** Characteristics of the studies on mortality prediction models for sepsis.

Study no.	Mortality prediction model	Analysis			
		ROC curve	Association	Correlation	Survival analysis
1	Day-1 PELOD-2 and day-1 “quick” PELOD-2 (qPELOD-2), pSOFA, P-MODS Zhong et al. (18)	PELOD-2, cut-off: 6.5 AUC: 0.916; 95%CI 0.888–0.938 qPELOD-2, cut-off: 1 AUC: 0.802; 95%CI 0.765–0.836 pSOFA, cut-off: 7.5 AUC: 0.937; 95%CI 0.913–0.957 P-MODS, cut-off: 3 AUC:0.761; 95%CI 0.722–0.798			
2	Mortality risk model for pediatric sepsis Chen et al. (2)	Training group, cut-off: 0.22462 AUC 0.854 L; 95%CI 0.826–0.881 Sensitivity 85.7%, specificity 70.1% Validation group, cut-off: 0.189165 AUC 0.844; 95%CI 0.816–0.873 Sensitivity 87.3%, specificity 67.7%	BNP > 7.1: OR 1.996; 95%CI 1.45–2.747; *p* < 0.001 Albumin < 3.5: OR 1.649; 95%CI 1.098–2.477; *p* = 0.016 Total bilirubin > 6: OR 2.3; 95%CI 1.45–3.658; *p* < 0.001 D-dimer positive: OR 2.921; 95%CI 2.078–4.108; *p* < 0.001 Mechanical ventilation in 24 h: OR 8.272; 95%CI 5.434–12.592; *p* < 0.001 Lactate > 2: OR 1.556; 95%CI 1.061–2.282; *p* < 0.024		
3	PRISM, PRISM III, PRISM IV, PIM, PIM2, PIM3, PELOD, PELOD 2 Niederwanger et al. (19)	PRISM: AUC 0.6; 95%CI 0.49–0.72 PRISM III: AUC 0.74; 95%CI 0.65–0.82 PRISM IV: AUC 0.69; 95%CI 0.59–0.79 PIM: AUC 0.76; 95%CI 0.67–0.85 PIM2: AUC 0.75; 95%CI 0.66–0.85 PIM3: AUC 0.71; 95%CI 0.6–0.82 PELOD: AUC 0.69; 95%CI 0.58–0.8 PELOD2: AUC 0.73; 95%CI 0.62–0.83 PRISM CRP: AUC 0.66; 95%CI 0.54–0.79 PRISM III CRP: AUC 0.81; 95%CI 0.73–0.89 PRISM IV CRP: AUC 0.8; 95%CI 0.72–0.88 PIM CRP: AUC 0.77; 95%CI 0.67–0.87 PIM2 CRP: AUC 0.77; 95%CI 0.67–0.87 PIM3 CRP: AUC 0.73; 95%CI 0.61–0.85 PELOD CRP: AUC 0.69; 95%CI 0.58–0.79 PELOD2 CRP: AUC 0.84; 95%CI 0.77–0.91			
4	Vasoactive-Inotropic Score McIntosh et al. (21)			Correlation analysis with ICU LOS and ventilator days	
5	DIC score Slatnick et al. (22)	1-year mortality, cut-off: 3; AUC 0.69 Sensitivity 0.7, specificity 0.62	30-day mortality: OR 2.99; 95%CI 0.54–16.6; *p* = 0.21 90-day mortality: OR 3.57; 95%CI 0.90–14.09; *p* = 0.07 1-year mortality: OR 3.72; 95%CI 1.48–9.35; *p* = 0.005		1-year mortality: HR 3.55; 95%CI 1.46–8.64; *p* = 0.005
6	PRISM score El-Nawawy (23)				The cut-off point of survival was a PRISM score of 26 with an expected/observed ratio of 1.05 for non-survivors with 91.6% accuracy
7	pSOFA score El-Mashad et al. (25)	pSOFA, cut-off 6.5 AUC 0.886; 95%CI 0.84–0.931; *p* < 0.0001 Sensitivity 80.9%, specificity 81.8%			
8	PRISM, PELOD El-Hamshary et al. (26)	PRISM III, cut-off: 20 AUC: 0.726; 95%CI 0.661–0.790 Sensitivity 63.8%, specificity 67.1% PELOD, cut-off: 13 AUC 0.788; 95%CI 0.729–0.846 Sensitivity 70.2%, specificity 69.9%			
9	Age-adjusted quick SOFA Van Nassau et al. (5)	qSOFA score, cut-off: 2 AUC: 0.72; 95%CI 0.57–0.86 Sensitivity 50, specificity 93.3%, NPV 98%, PPV 22.5%			
10	Modified PRISM-III Leon et al. (34)	Modified PRISM III score, cut-off: 13 Sensitivity 71%, specificity 64%			
11	PRISM-III-APS Pollack et al. (37)	PRISM III-APS training AUC:0.95 ± 0.007 PRISM III-APS validation AUC: 0.902 ± 0.027			
12	Age-adapted SOFA Wu et al. (38)	In-hospital mortality, cut-off: 2 Crude AUROC: 0.757; 99%CI 0.715 – 0.789; *p* < 0.001 Adjusted AUROC: 0.771; 99%CI 0.732 – 0.81; *p* < 0.011			

PELOD-2, performance of the pediatric logistic organ dysfunction; pSOFA, pediatric sequential organ failure assessment; AUC, area under the curve; CI, confidence interval; BNP, B-type natriuretic peptide; OR, odds ratio; PRISM, pediatric risk of mortality; PIM, pediatric index of mortality; DIC, disseminated intravascular coagulation; pSOFA, pediatric sequential organ failure assessment; HR, hazard ratio.

**Table 6:** Characteristics of the studies of mortality predictors in sepsis.

Study no.	Mortality prediction model	Analysis			
		ROC curve	Association	Correlation	Survival Analysis
1	Bioelectrical impedance phase angle Zamberlan et al. (14)	PA, cut-off 2.8° AUC: 0.65; 95% CI, 0.58–0.71 Sensitivity 37.1%, specificity 86%			
2	Crystalloid fluid administration over 3 days Zhang et al. (16)		High crystalloid with PICU mortality (*p* < 0.041)		
3	Immunology markers Ibrahiem et al. (27)	NK cell concentration, cut-off 10 AUC: 0.95; 95% CI, 0.889–1.0; *p* < 0.001 Sensitivity 100%, specificity 86%, PPV 70%, NPV 100%, accuracy 89.5%			
4	Reduction in procalcitonin level Poddar et al. (29)	The number of deaths was too small to provide a good estimate of the area under the ROC curve for a reduction in PCT level to predict survival. However, an absolute decrease of PCT of ≥4 ng/ml or a percentage reduction of ≥50% in the first four days of ICU stay predicts survival with a sensitivity of 78% and specificity of 83%			
5	Plasma mtDNA level Yan et al. (30)	Plasma mtDNA, cut-off: 890.43 AUC: 0.726; *p* < 0.0001 Sensitivity 88.5%, specificity 53.6%			
6	Urinary L-FABP Yoshimatsu et al. (31)	L-FABP first urine, cut-off: 370 ng/ml AUC: 0.663; 95%CI0.455–0.871 Sensitivity 75%, specificity 66.7% L-FABP day 2, cut-off: 580 ng/ml AUC: 0.809; 95%CI 0.612–1,0 Sensitivity 81.8%, specificity 90% L-FABP first urine, cut-off: 2275 mcg/g creatinine AUC: 0.675; 95%CI 0.463–0.886 Sensitivity 75%, specificity 66.7% L-FABP day 2, cut-off 1570 mcg/g creatinine AUCL 0.85; 95%CI 0.666–1,0 Sensitivity 90.9%, specificity 85%			
7	Vascular reactivity index Lee et al. (33)	VRI-24 h, cut-off: 50 AUC: 0.83; *p* = 0.007 Sensitivity 82%, specificity 75%, LR + 3.3, LR − 0.2; Youden index 0.6 VRI-48 h, cut-off: 61 AUC 0.81; *p* = 0.033 Sensitivity 71%, specificity 82%, LR + 2.9, LR − 0.3; Youden index 0.5			
8	Thiol-disulphide homeostasis Ayar et al. (35)				
9	Macrophage migration inhibitory factor (MIF) Emonts et al. (36)		MIF levels were significantly higher in non-survivors At the entry, *p* < 0.001 At 12 h, *p* = 0.005 At 24 h, *p* = 0.01		

ROC, receiver operating characteristic; PICU, pediatric intensive care unit; NK, natural killer; AUC, area under the curve; L-FABP, liver-type fatty acid binding protein; PA, phase angle; PCT, procalcitonin; VRI, Vascular Reactivity Index; LR = likelihood ratio.

### Characteristics of the mortality prediction models

The characteristics of the mortality prediction models are presented in Tables 1, 5–7. Out of 28 prediction models, 19 were developed prospectively, while 11 used retrospectively collected data. The study durations varied from 4 months to 9 years. Three studies did not specify the study duration or data collection time (34, 36, 39). One study included both children and adult patients, and one study was multi-center (36). Eight studies did not specify the ages of their patients. Seven studies were done in developed countries (5, 19, 22, 35–37, 39). These studies included several parameters that are typically unavailable in developing countries, including pancreatic stone protein, macrophage migration inhibitory factors, plasma mitochondrial DNA, and metabolomic studies (28, 30, 36, 39). The number of included patients in each study ranged from 25 to 11,163. Several studies limited the participants to all patients admitted to the PICU (16, 23, 26–28, 30, 36, 37).

**Table 7:** Characteristics of the studies of mortality prediction models and predictors in sepsis.

Study no.	Mortality prediction model	Analysis			
		ROC curve	Association	Correlation	Survival analysis
1	Troponin T and I, PELOD 2 Dauhan et al. (15)	Troponin T, cut-off 40.3 ng/ml AUC 86.4%; 95%CI 0.75–0.97; *P* < 0.001 Sensitivity 76%, specificity 75% Troponin I, cut-off 0.125 ng/ml AUC 92.6%; 95%CI 0.85–1.0; *p* < 0.001 Sensitivity 80%, specificity 81.3%		Troponin T-24 h: *r *= 0.137; p0.394 Troponin T-48 h: *r* = 0.771; p < 0.001 Troponin I-24 h: *r* = 0.326; p = 0.037 Troponin I-48 h: *r* = 0.691; *p* < 0.001	
2	Serum albumin Kim et al. (17)	Albumin: AUC 0.702; 95%CI 0.633–0.772 PIM 3 + Albumin: AUC 0.82; 95%CI 0.766–0.874 PRISM III + Albumin AUC: 0.857; 95%CI 0.81–0.904	Hypoalbuminemia with 28-mortality rate (*p* < 0.001)		
3	Lactate level Jat et al. (20)	PRISM III score, cut-off 10 AUC 0.909; 95%CI 0.802–1.016; *p* < 0.0001 PPV 70%, NPV 90% Lactate 1(0–3 h), cut-off 5 mmol/L AUC 0.786; 95%CI 0.596–0.975; *p* = 0.014 PPV 38%, NPV80% Lactate 2(12 h), cut-off 5 mmol/L AUC 0.792; 95%CI 0.597–0.986; *p* = 0.012 PPV 71%, NPV 83%, Lactate 3(24 h), cut-off 5 mmol/L AUC 0.786; 95%CI 0.580–0.991; *p* = 0.023 PPV 64%, NPV 83%	PRISM III score, cut-off 10 OR 21; 95%CI 2.155–204.614; *p* = 0.002 Lactate 1(0–3 h), cut-off 5 mmol/L OR 6.7; 95%CI 1.047–42.431; *p* = 0.034 Lactate 2(12 h), cut-off 5 mmol/L OR 12.5; 95%CI 1.850–84.442; *p* = 0.005 Lactate 3(24 h), cut-off 5 mmol/L OR 8.6; 95%CI 1.241–61.683; *p* = 0.021		
4	Serum procalcitonin, serum albumin, PEWS Xie et al. (24)	PCT, cut-off: 59.65 mcg/L AUC: 0.73 Sensitivity 53.2%, specificity 85.1% Albumin, cut-off: 3.52 g/dl AUC: 0.761 Sensitivity 57.45%, specificity 85.11% PEWS, cut-off 6.5 points AUC 0.771 Sensitivity 74.5%, specificity 68.1% PCT, albumin, PEWS combination AUC: 0.908 Sensitivity 87.23%, specificity 85.11%			
5	High-sensitivity C-reactive protein, serum procalcitonin, pancreatic stone protein Wu et al. (28)	hsCRP, cut-off: 76.1 mg/ml AUC: 0.76; 95%CI 0.70–0.82; *p* < 0.01 Sensitivity 87.3%, specificity 60.7% PCT, cut-off 47 ng/ml AUC: 0.83; 95%CI 0.77–0.88; *p* < 0.01 Sensitivity 72.1%, specificity 68.1% PSP, cut-off 256 ng/L AUC: 0.73; 95%CI 0.67–0.79; *p* < 0.01 Sensitivity 79.7%, specificity 57.7% PCT, CRP, PSP: AUC 0.92; 95%CI 0.87–0.95; *p* < 0.001 Sensitivity 73.4%, specificity 93.3%			
6	Thrombomodulin Khattab et al. (32)	Thrombomodulin, cut-off: 5.0 AUC 0.711; 95%CI 0.569–0.847; *p* = 0.118 Sensitivity 80%, specificity 80%, PPV 24%, NPV 98%, accuracy 80% PRISM, cut-off 4.25 AUC: 0.918; 95%CI 0.819–1.0; *p* = 0.002 Sensitivity 80%, specificity74%, PPV 69%, NPV 98%, accuracy 74% PIM, cut-off: 6.8 AUC: 0.96; 95%CI 0.91–1.0; *p* = 0.001 Sensitivity 100%, specificity 86%, PPV 88%, NPV 100%, accuracy 87%			
7	Metabolomics approach Mickiewicz et al. (39)	Metabolomics AUC 0.91; sensitivity 80%, specificity 90%, PPV 89%, NPV 82%, accuracy 85% PRISM III-APS AUC: 0.85; sensitivity 70%, specificity 80%, PPV 78%, NPV 73%, accuracy 75% Orthogonal partial least squares discriminant analysis models 1st model: septic shock specimens AUC 0.91, *p* = 0.0044 2nd model: septic shock specimen with a complicated course AUC 1.0, *p* = 0.00043			

ROC, receiver operating characteristic; AUC, area under the curve; PELOD, performance of the pediatric logistic organ dysfunction; PIM, Pediatric Index of Mortality; PRISM, pediatric risk of mortality; OR, odds ratio; PEWS, Pediatric Early Warning Score; PCT, procalcitonin; PPV, positive predictive value; NPV, negative predictive value; PSP, pancreatic stone protein; CRP, C-reactive protein.

### Outcomes measured

The timing of mortality outcomes varied between studies. Nevertheless, only eight studies specified the timing of mortality, and two had mortality as a secondary outcome (21, 22). Most studies did not specify the timing of mortality. Nine studies reported secondary outcomes such as length of hospital/PICU stay, ventilator requirement, or vasoactive agent usage (5, 16, 21, 22, 25, 32, 38). Mortality rates ranged between 4.8%and 64%. Missing data were excluded in seven studies (2, 5, 16, 18, 20–22, 31, 39). Only one study entered the missing data as normal values (38).

## Discussion

The predictive value of each mortality model is shown in Table 2.

PELOD-2 had good predictive power. Estimating PELOD-2 at day 1 of admission had a high area under the curve (AUC) (0.916; 95% CI, 0.888–0.938). Even with some modification of PELOD-2, its AUC was still 0.802 (95% CI, 0.765–0.836) (18). Combining PELOD-2 with C-reactive protein (CRP) increased its predictive value to 0.84 (95% CI, 0.77–0.91) (19). These findings showed a better predictive value for PELOD-2 compared to PELOD. The SOFA score returned similar results. The predictive value of pSOFA was similar to that of PELOD. pSOFA measured at day 1 also had a high AUC (0.937; 95% CI, 0.913–0.957) (18). Its AUC showed good predictive value even with modifications such as qSOFA and age-adapted SOFA (with AUCs of 0.72 and 0.771, respectively) (5, 25). PRISM-III had lower predictive power than PELOD-2. Overall, the studies had a PRISM-III predictive power above 0.7 (19, 26, 34). A modification of PRISM-III based on physiologic criteria (PRISM-III-APS) increased its predictive value to 0.85–0.95 (38, 39).

The VIS had a strong independent association with death. For every unit increase of VIS at 12 h, there was a 14% increase in the odds of subsequently experiencing the composite outcome (*p* < 0.001). This finding was independent of the measured Pediatric Index of Mortality-3 (PIM3) score (21).

The Vascular Reactivity Index, defined as a systemic vascular resistance index subdivided by VIS (SVRI/VIS) measured at hour 0 in children with persistent refractory shock, had an AUC of 0.85—the highest measured (95% CI, 0.65–0.95; *p* = 0.001)—for predicting 28-day mortality when administered during the first 72 h. A VRI <18 at 0 h had 100% specificity for predicting mortality. The best cutoff values of the VRI increased from more than 30 at 0 h–12 h to more than 60 at 30 h–48 h. Most children with a cutoff VRI below 30 had a 100% likelihood of mortality, even after aggressive resuscitation, whereas most of those with a VRI >80 at 0 h–18 h and >100 at 24 h–48 h had the highest likelihood of survival (sensitivity 100%) (33).

Crystalloid fluid administration is associated with mortality in pediatric patients with severe sepsis or septic shock. The high crystalloid group (>193 ml/kg body weight) had a higher PICU mortality (46.2% vs. 25%; odds ratio [OR] 2.57; 95% CI, 0.99–6.67; *p* = 0.041) compared to the low crystalloid group (16).

Albumin had an excellent predictive value when combined with other variables. Albumin alone had a predictive power of 70.2%–76.1%. When combined with PIM3 and PRISM-III scores, the predictive power increased to 82% and 85.7%, respectively (17). When combined with other variables, such as B-type natriuretic peptide (BNP), total bilirubin, D-dimer, mechanical ventilation, and lactate, it had an increased predictive value, with an AUC in the range of 84.4%–85.4% (2). The highest recorded predictive power of albumin, 90.8%, was in combination with serum procalcitonin and the Pediatric Early Warning Score (PEWS).

Serum procalcitonin alone had a predictive value of 73%–83% (24, 28). When combined with serum albumin and PEWS, the predictive power increased to 90.8% (24). In addition, when serum procalcitonin was combined with high-sensitivity C-reactive protein (hsCRP) and pancreatic stone protein, the predictive power increased to 92% (28). Other studies investigated the predictive power of a reduction in procalcitonin levels for sepsis mortality. Nevertheless, an estimation could not be made because of the low number of outcomes (29). Troponin I had the highest predictive power (AUC 92.6%) compared with other biomarkers, but only a few centers were able to evaluate it; its highest predictive power was at 48 h (15).

Lactate had the greatest association with mortality. Lactate levels above 5 mmol/L had their highest predictive power (AUC 79.2%; 95% CI, 0.597–0.986) and association with mortality (OR 12.5; 95% CI, 1.85–84.442; *p* = 0.005) when measured at 12 h (20). The immediate measurement of the lactate level was more associated with mortality when using a higher cutoff (2 mmol/L [OR 1.556; 95% CI, 1.061–2.282; *p* < 0.024] vs. 5 mmol/L [OR 6.7; 95% CI, 1.047–42.431; *p* = 0.034]) (2, 20). Slatnick et al. found that a DIC score ≥3 predicted an increased mortality risk for up to 1 year, with a hazard ratio (HR) of 3.55 (95% CI, 1.46–8.64; *p* = 0.005). It was slightly higher than that of the lactate level measured within 24 h of admission (HR 3.03; 95% CI, 1.28–7.72; *p* = 0.012). Moreover, the DIC score had a predictive power with an AUC of 69% in predicting 1-year mortality (22).

Several biomarkers also had a predictive value for sepsis mortality, such as first urine liver-type fatty acid binding protein (L-FABP), natural killer (NK) cell concentration, and serum thrombomodulin. The diagnostic performance of the first urine L-FABP was analyzed using the receiver operating characteristic (ROC) curve, and it was found to have an AUC of 0.647 (95% CI, 0.500–0.795) (31). The relative concentration of NK cells (CD3–CD56/16+%) at day 1 had a significant predictive ability (*p* < 0.001) to detect mortality (AUC 0.950; 95% CI, 0.889–1.0) (27). In addition, the serum thrombomodulin level had an AUC of 0.711 for predicting mortality (31). Other biomarkers, such as plasma mtDNA, phase angle value, macrophage migration inhibitory factor, and plasma thiol-disulfide, also showed a significant association and positive correlation with mortality (14, 30, 35, 36).

A tool with a discriminatory ability of 0.80 (AUC) or more was identified as good for discrimination. The closer the ROC curve area was to 1.0, the better the prediction model. Modified prediction models, i.e., the pSOFA (0.937), PELOD-2 at day 1 admission (0.916), and the Pediatric Risk of Mortality-III-Acute Physiology Score (PRISM-III-APS) (0.85–0.95), met these benchmarks, indicating that these three tools can discriminate between survival and non-survival in pediatric patients, primarily PRISM-III in combination with other predictors such as albumin (predictive power increased to 85.7%) (17).

All studies were assessed for risk of bias by evaluating the calibration and discrimination using the Hosmer–Lemeshow goodness-of-fit test and concordance index. However, one study showed a high risk of bias due to inappropriate analysis, no clear inclusion and exclusion criteria, and its handling of missing data (23). A total of 15 models had similar characteristics to their participants and matched predictors and outcomes with the research question.

Within the prediction models using biomarkers, serum albumin is highly applicable in acute care in resource-limited settings. Acute care refers to secondary healthcare, where a patient receives active but short-term treatment of sepsis in the emergency department or PICU. Furthermore, serum albumin evaluation was widely available and cost less than other biomarkers. Serum albumin had the best predictive power compared to other biomarkers, especially when combined with other predictors, such as serum procalcitonin and PEWS (AUC 90.8%, sensitivity 87.23%, specificity 85.11%) (24).

Serum procalcitonin had an even higher predictive power (AUC 92%) when combined with hsCRP and pancreatic stone protein. However, the study examining it showed a high risk of bias due to unclear participant selection and analysis (28). Rarer biomarkers, such as NK cell concentration, were still applicable in our setting, even though they are not widely used or available. The study evaluating NK cell concentration as a prediction model was considered to have a low risk of bias, even with a small sample size, because of its clear participant selection, predictor, and outcome. In addition, the discrimination value of NK cell evaluation was considered suitable due to the high AUC (95%; 95% CI 0.889–1.0) (27).

Lactate, a biomarker widely measured in patients with sepsis, was highly associated with mortality. It was applicable due to being widely available in numerous health care facilities. However, its predictive value is lower compared with other biomarkers; it therefore might be better to evaluate mortality with predictors with better discrimination, such as serum albumin. The bioimpedance phase angle was not considered applicable in our setting. Nevertheless, the study also included measurements using more straightforward methods, such as the mid-upper arm circumference, which was feasible in numerous health care facilities (14).

One of the limitations of this study was the study selection. Only studies in English were eligible for analysis. In addition, some prediction models did not have an AUROC analysis, so the predictive power was more challenging to determine. Despite its limitations, lactate is an easily measured laboratory parameter that can provide helpful information for the bedside clinician when incorporated into the appropriate clinical context. Thus, it is essential to interpret lactate cautiously, as its reported level can be due to tissue hypoperfusion, decreased lactate clearance, or use of epinephrine.

The strength of our study lies in the fact that it is the first scoping review to investigate the prognostic models and predictors that are available in developing countries.

## Conclusion

Leveraging prognostic models to predict mortality among pediatric patients with sepsis remains an important and well-recognized area of study. While much validation and development work remains to be done, available prognostic models could aid clinicians at the bedside of children with sepsis. Furthermore, mortality prediction models are essential and valuable tools for assessing the quality of care provided to critically ill pediatric patients. In the future, these models should be prospectively validated and refined across diverse patient populations.

## Data Availability

The original contributions presented in the study are included in the article/Supplementary Material, further inquiries can be directed to the corresponding author.
